# Variable Link Performance Due to Weather Effects in a Long-Range, Low-Power LoRa Sensor Network

**DOI:** 10.3390/s21093128

**Published:** 2021-04-30

**Authors:** Thomas Ameloot, Patrick Van Torre, Hendrik Rogier

**Affiliations:** IDLab, Department of Information Technology (INTEC), Ghent University-IMEC, Technologiepark-Zwijnaarde 126, B-9052 Ghent, Belgium; patrick.vantorre@ugent.be (P.V.T.); hendrik.rogier@ugent.be (H.R.)

**Keywords:** Internet of Things, LoRa, LPWANs, tropospheric radiowave propagation, wireless sensor networks

## Abstract

When aiming for the wider deployment of low-power sensor networks, the use of sub-GHz frequency bands shows a lot of promise in terms of robustness and minimal power consumption. Yet, when deploying such sensor networks over larger areas, the link quality can be impacted by a host of factors. Therefore, this contribution demonstrates the performance of several links in a real-world, research-oriented sensor network deployed in a (sub)urban environment. Several link characteristics are presented and analysed, exposing frequent signal deterioration and, more rarely, signal strength enhancement along certain long-distance wireless links. A connection is made between received power levels and seasonal weather changes and events. The irregular link performance presented in this paper is found to be genuinely disruptive when pushing sensor-networks to their limits in terms of range and power use. This work aims to give an indication of the severity of these effects in order to enable the design of truly reliable sensor networks.

## 1. Introduction

Since many Internet of Things (IoT) applications rely on the deployment of low-power wireless sensor networks (WSNs) over rather large areas, research on sub-GHz wireless communication technologies has seen a steady rise in popularity. Modern low-power, wide-area network (LPWAN) technologies such as NarrowBand IoT (NB-IoT) [1], Long Term Evolution-Machine Type Communication (LTE-M) [2], Dash7 [3], SigFox [4] and, in particular, “Long Range” (LoRa) [5] receive a lot of attention, as they trade in data rate for communication range, link reliability and power efficiency. Therefore, they could soon become ubiquitous in the low data-rate sensor networks, part of the fifth generation of mobile communication technologies (5G).

LoRa technology has been assessed in a relatively large number of publications. First of all, general descriptions of operational aspects of LoRa technology are presented in [6,7,8,9]. More in-depth, theoretical reviews of LoRa modulation are found in [10,11]. The physical-layer performance of LoRa is discussed for indoor environments in [12,13,14,15,16,17,18]. Outdoor measurement campaigns are presented in [19,20,21,22,23,24,25]. When pushing the boundaries of LPWAN technologies, such as LoRa, by deploying outdoor sensor networks with relatively large inter-nodal distances, large-scale propagation effects and mechanisms such as obstruction fading, tropospheric scattering and tropospheric ducting may become increasingly important as these may have a sizeable impact on the quality and reliability of wireless links [26]. Given that both tropospheric scattering and tropospheric ducting could vastly increase the communication range for a single wireless link [27,28], inter-cell interference may turn out to be just one of those hurdles when scaling up modern LPWAN technologies. In case of LoRa, [6,7,29,30] have actually warned for reduced performance when the number of end-devices grows. Furthermore, adverse weather conditions are known to impact outdoor antenna performance and electronic circuit reliability. In general, it is important to identify the challenges these effects pose to engineers when designing sensor networks spanning large distances. However, to this date, the amount of literature on the impact of large-scale propagation phenomena and weather effects in LoRa networks is limited.

### 1.1. Related Work

For LoRa technology, research indicates that temperature variations can have a significant impact on the wireless links [31,32]. Ref. [31] describes different experiments where large packet loss is recorded when the node temperature is very high. A small hysteresis is observed in moments when the receiver is warming up or cooling down. In outdoor experiments, correlation between temperature, humidity, packet reception rate, and received signal strength is observed. Similarly, in [32], the authors present an experimental evaluation of the reliability of LoRa receivers in the presence of temperature variations. Lab measurements are performed, which show that for each increase of 10 ${}^{\circ }C$ in ambient temperature, the received signal level is lowered by approximately 1 dB. Additionally, a detailed investigation is carried out that demonstrates how selecting the right LoRa settings may increase the probability of packet reception. Finally, design guidelines are provided to alleviate the impact of temperature effects on link quality.

Recently, a number of long-term measurement campaigns that monitor link quality over time have been published. In [33], received signal levels are presented for outdoor sensors deployed in the north of Sweden. It is observed that noise power is reduced when temperatures are low, which leads to better signal-to-noise ratios (SNRs). The authors of [33] also demonstrate that snowfall has a detrimental impact on link quality, especially when large distances are covered. To assess LoRa performance for flooding prevention, [34] presents an in-depth analysis of LoRa propagation characteristics in different land and water environments. Different antenna heights, node distances and, where applicable, tidal water levels are considered. The authors of [34] conclude that for over-water links, the communication distance and reliability are significantly affected by tides when the nodes are placed at low heights. Furthermore, they present a battery lifetime estimation model, which is very valuable for network planning and maintenance. Similarly, [35] presents received power levels from a 8.33 km link over water, employed to share data on offshore breeding cages in a fish farming plant. Sensor data on environmental parameters, such as temperature, relative humidity and atmospheric pressure, as well as weather conditions and marine parameters are gathered and compared to the measurements, which were gathered over a period of 70 days. In [35], link variations are observed as a result of changes in relative humidity, as well as due to the presence of rain. However, the link fluctuations presented are relatively small. It is mentioned that these may be larger on longer oversea link paths.

In a multitude of other publications, LoRa networks are employed to communicate sensor data describing the ambient temperatures, relative humidity levels or other meteorological parameters [36,37,38]. However, none of these papers compare the measured parameters to the performance of the network. Conversely, a large number of papers have been published that discuss temperature and/or humidity effects in WSNs in general, but which do not employ LoRa technology [39,40,41,42,43,44,45,46,47]. In all of these publications, it is shown that received signal levels and packet reception are both lower when temperatures are higher. Yet, once more, the impact on the received signal strength is limited to a number of decibel for very large temperature swings. In [41], it is stated that links at the edge of the communication range are the most impacted. Interestingly, [47] shows that high relative humidity values may have an impact on signal strengths, particularly when ambient temperatures are low (below 0 ${}^{\circ }C$).

### 1.2. Contributions

This work aims to contribute to existing LoRa network monitoring research by analysing the prevalence and severity of link fluctuations in LPWANs through the analysis of channel monitoring data gathered from an actual LoRa network over a timespan of multiple months. This WSN was established by deploying six custom-built wireless nodes, specifically designed for LoRa channel characterisation, at various locations in and around the city of Ghent, Belgium. The paper is structured as follows. First, in Section 2, the wireless sensor network setup used to gather the data presented in this work is described. Subsequently, in Section 3, the gathered experimental data are presented, revealing large signal deteriorations measured daily along the longest links in the network. In Section 3.2, Section 3.3, Section 3.4 and Section 3.5, an evidence-based approach is adopted to describe and identify the nature of these fluctuations. Additionally, some potential consequences of these effects with respect to LPWANs are highlighted. Finally, in Section 5, a conclusion to this work is presented.

## 2. Materials and Methods

In order to examine the performance of IoT sensor networks, measurement data were gathered from an actual LoRa network in the city of Ghent, Belgium. This network was established using custom LoRa nodes, developed specifically for channel characterisation purposes and thoroughly described in [17]. In this section, a very brief outline of the characteristics and functions of this hardware is given, together with the deployment details of the network and the measurement methodology adopted to gather the data.

### 2.1. Hardware

**Figure 1:**
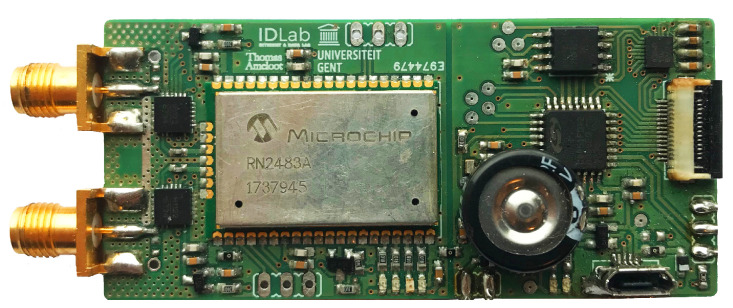
PCB-implementation of the custom LoRa channel characterization hardware [17].

In short, the custom LoRa transceiver system used in this research is built around a low-power, 8-bit microprocessor and a dual-frequency LoRa transceiver module, facilitating communication in both the 434 MHz and 868 MHz ISM-bands. It also includes some peripheral hardware such as a real-time clock (RTC), an inertial measurement sensor unit (IMU) and a 32 Mbit flash memory IC to locally store measurement data. Additionally, attenuators are used to significantly increase the dynamic range for SNR measurements. Using these off-the-shelf components, this hardware is much more comparable to what one would expect to find in actual LoRa applications, yielding more realistic system performance results. Furthermore, this hardware is a lot smaller, cheaper and more energy-efficient than channel-sounding equipment, enabling deployment at locations where it would not be feasible to install such bulky and expensive lab equipment. Figure 1 shows a compact ( 72 mm× 30 mm) PCB-implementation of this custom LoRa transceiver.

**Figure 2:**
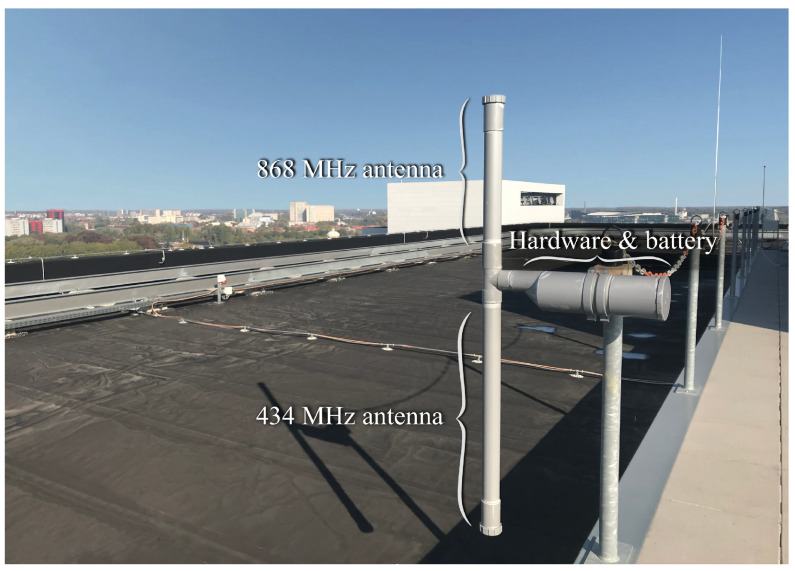
Outdoor setup of one of the LoRa nodes.

In most receiver locations, the transceiver system shown in Figure 1 was paired with an end-fed half-wavelength dipole, impedance-matched via a quarter-wave stub [48,49]. For each LoRa transceiver, two of these antennas were manually fabricated, the first one intended for use in the 434 MHz band and the second one intended for use in the 868 MHz band. For each of the deployed antennas, the correct operation was verified in the lab. H-plane radiation patterns are omnidirectional with a gain of 2.2 dBi. For nodes to be deployed outdoors, waterproof polyvinyl chloride (PVC) enclosures were produced that fit both the 434 MHz and 868 MHz antennas along with the LoRa hardware and a large-capacity battery. A deployment of one of these setups can be seen in Figure 2. Owing to the low power consumption of the LoRa node, such a basic outdoor setup can operate autonomously for multiple months on end.

### 2.2. Node Deployment

**Figure 3:**
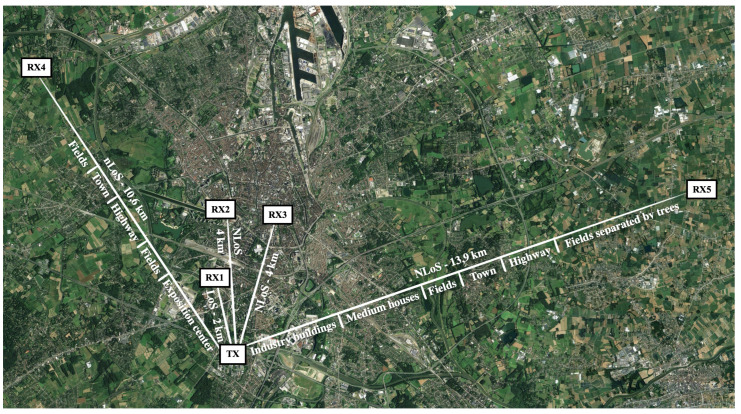
LoRa network node locations in and around the city of Ghent, Belgium. Map Data: Google, Landsat/Copernicus.

When gathering the measurements presented in this work, the LoRa nodes were configured to form a broadcast network, as this enables reliable time-synchronisation between the nodes. The transmitter (TX), which is shown in Figure 2, was placed at a height of approximately 55 m, on the roof of a modern 12-story office building in the south of the city of Ghent. The locations of the receiving nodes are shown in Figure 3. This figure also includes descriptions of the different types of obstructions in the longest link paths. These obstructions mostly consist of buildings or trees as the area surrounding the city of Ghent is largely flat. Hence, there are no large geographical features such as hills or mountains which significantly obstruct the link paths. Each of the receiver locations is considered in more detail below.

#### 2.2.1. RX1

The first receiver was deployed on the second floor of a university building, located at 2.1 km from the transmitter. The propagation path between TX and RX1 is mostly urban, as it encompasses multiple large, low-rise buildings and some larger road infrastructure. Although the RX1 receiving node was deployed indoors, this path can be labeled Line-of-Sight (LoS) as the only obstructions between the transmitting and receiving antennas were the glass window in front of the two receive antennas and the PVC enclosure covering the two transmit antennas, which had very little influence on the performance of these antennas.

#### 2.2.2. RX2

A second receiver was deployed on the fifth floor of another university building, located at 4.0 km from the transmitter. The path from TX to RX2 includes the very same features as the path to RX1, in addition to several high-rise apartment buildings, located closer to RX2. As a consequence, the path between TX and RX2 is Non-Line-of-Sight (NLoS), although the receive node was again located close to a window. This implies that, for this receiver, the reception of LoRa packets depends on signal diffraction, reflection and potential transmission through these structures.

#### 2.2.3. RX3

As for the TX-RX2 link, the path between TX and RX3 is NLoS due to the surrounding buildings. Yet, RX3 is a fully urban receiver location, located in the heart of the city of Ghent, also at 4.0 km from the transmitter. However, in this environment, the buildings are a lot lower than the apartment towers surrounding RX2. Additionally, this receiver was deployed at a lower altitude than the one at RX2, being on the third floor of a laboratory building. It is important to note that the direct path between TX and RX3 is also obstructed by a large medieval abbey. Consequently, its very thick stone walls may have a measurable influence on the average power received at this location.

#### 2.2.4. RX4

The fourth receiver was installed in the attic of a house in a suburban satellite town located to the northwest of the city. At a distance of 10.6 km to the transmitter, this link is significantly longer than the previous ones. Nevertheless, as the direct path to this link is mostly unobstructed by buildings and as there are no large geographical features in the surrounding area, an acceptable signal level can still be expected. Although no large obstacles are present, this path does contain a lot of vegetation and some residential areas. As the trees (western European broad-leaves trees: 10 to 15 m tall) are generally higher than the houses (two or three floor levels: 6 to 10 m high) in this suburban area, these are considered to be the most important features affecting the wireless link. Yet, since trees are not really solid structures, the radio link is classified near-line-of-sight (which is denoted here as nLoS). Eventually, to increase the link budget and, hence, the packet reception ratio (PRR), two Yagi-Uda antennas were used at RX4. These antennas, aligned with the transmitter, exhibit gains of 9 dBi and 13 dBi at 434 MHz and 868 MHz, respectively.

#### 2.2.5. RX5

Similar to the fourth receiver, the fifth receiver was also deployed in the attic of a house. However, this house is located further away from the transmitter, to the east of the city of Ghent, where the environment is more rural. Although this 13.9 km radio link between TX and RX5 is mostly rural, closer to the transmitter, there are some office buildings and a large football stadium, both of which may obstruct the direct propagation path, making the categorisation as NLoS the most accurate for this link. Similar to the receiver at RX4, two Yagi-Uda antennas were used to increase the PRR.

#### 2.2.6. TX Monitor

A receiver was also placed in a technical room, on the same roof as the transmitter. This receiver was used to monitor the stability of the transmitter’s power output.

### 2.3. Link Models

**Figure 4:**
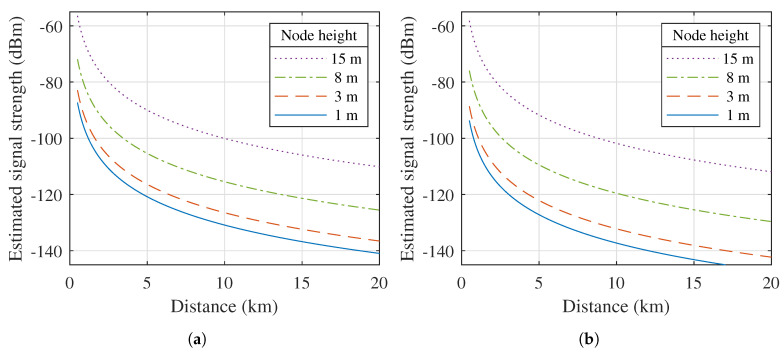
Estimated signal strength received by a remote node at different heights, based on the Okumura-Hata model [50]. (Transmit power = 10 dBm, transmitter height = 55 m.) (**a**) 434 MHz. (**b**) 868 MHz.

It is interesting to assess the expected performance of each link presented above. To this end, the Okumura-Hata propagation model [50] was employed to estimate the signal strengths received by the remote nodes as a function of the distance between each receiver and the transmitter. These signal strengths are shown in Figure 4 for node heights of 1, 3, 8 and 15 m. This model provides an indication of the average powers that may be received when antenna placement is optimal and when there is a line-of-sight between the transmitter and the receiver. Yet, it is important to note that in practical IoT deployments, remote nodes are often placed in a suboptimal position for communication with the base station. In fact, whereas in other types of applications, the antenna might be deployed on a mast to achieve a reliable connection, WSN nodes are usually installed much closer to link obstructions, as is also apparent from the node placements described in Section 2.2.

Furthermore, given the long distances covered in the network, the first Fresnel zone is often populated with a large amount of scatterers and obstacles. As an illustration, the maximum radius $r_{1}$ of the first Fresnel zone is calculated for each link by applying the following expression:

(1) $$r_{1}=8.656\;\sqrt{\frac{d}{f}},$$

where *d* denotes the distance to the transmitter and *f* indicates the operating frequency [50]. We also calculate the minimum clearance for this zone when no obstacles are present, besides the curvature of the earth, which is taken into account through subtracting $H=375d^{2}/4R$ from the absolute Fresnel clearance, with *R* denoting the radius of the Earth. The resulting quantity, which is expressed as a percentage, is denoted as $c_{0}$ and is calculated through

(2) $$c_{0}=\frac{1}{r_{1}}\;\cdot \;\left(\frac{|h_{\mathrm{TX}}-h_{\mathrm{RX}}|}{2}-H\right)\;\cdot \;100\%,$$

where $h_{\mathrm{TX}}$ and $h_{\mathrm{RX}}$ indicate the heights of the transmitter and receiver, respectively. However, as mentioned earlier, there are a lot of obstacles present in the link paths described in Section 2.2. Both the maximum radii of the first Fresnel zones and the Fresnel zone clearances are provided for each link in Table 1. As can be deduced from the values for $r_{1}$, all of the links to RX2, RX3, RX4 and RX5 have their first Fresnel zone intersecting with the ground. Furthermore, one must also keep in mind that obstructions may further limit the Fresnel zone clearance by a significant margin. As a result, it can be expected that the signal levels received by the nodes may be a lot lower than those calculated by means of the Okumura-Hata model.

### 2.4. Measurement Methodology

As summarised in Table 2, 16-byte packets were broadcast that include a transmitter identifier string, a unique packet number and a timestamp as well as some information on the transmitter’s operational status, such as its power supply level (VDD) and the ambient temperature (TEMP).

Although the 434 MHz and 868 MHz bands used in the network described above are ISM-bands, there is a maximum duty cycle imposed by law to avoid users occupying the channel for too long. To comply with the legal duty cycle limitations, one packet was transmitted every minute, alternating between both bands. Hence, per day, 720 packets were transmitted in each of the bands. A full overview of the LoRa modulation and the general network settings are described in Table 3. A LoRa spreading factor of 12 is chosen as the most remote nodes in the network can only be reliably reached by using this setting, which is the highest configurable spreading factor, corresponding to the highest sensitivity. The bandwidth and code rate both assume their default values.

## 3. Measurement Results and Analysis

Based on the measurement setup described in the previous Section, various measurement campaigns were performed. The general performance of the network is discussed first. Then, the most interesting features in the data gathered from this network are investigated and thoroughly analysed.

### 3.1. General Results

**Figure 5:**
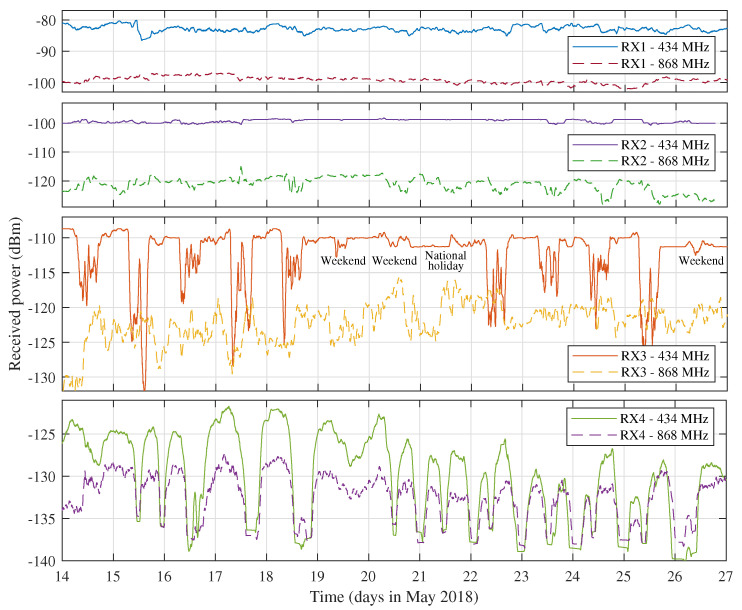
Selection of the general measurement results showing some of the most interesting features found in the datasets.

Figure 5 shows the power levels received by nodes RX1, RX2, RX3 and RX4, over a period of about two weeks in May 2018. On average, these are significantly lower than those estimated by the Okumura-Hata propagation model in Section 2.3. However, as discussed, this is to be expected when considering the specific placement of the nodes and the abundance of link obstructions in each link path. As a lot of WSNs are deployed in densely urbanised environments, average power levels may indeed vary greatly depending on the exact placement of each node. Consequently, it is the most interesting to assess the link variations with respect to the average received power in order to evaluate link quality. The curves plotted in Figure 5 clearly illustrate the different node behaviours found in the network. They were obtained by filtering the raw data using a one-hour moving average window to eliminate potential interference and reveal the underlying trends. This strategy was applied to all time-domain representations of received power level data presented in this work. The links to the first two receivers (RX1 and RX2) were found to be very stable. This is also reflected in Table 4, which shows the average received power levels (μ) and the standard deviations (σ) of these data. Additionally, the packet reception ratios (PRRs) for these links, which are also listed in Table 4, are very high.

Figure 5 and Table 4 also show that the standard deviation of the power measurements performed on the 434 MHz link to RX3 is significantly higher than the standard deviations measured for the 434 MHz links to RX1 and RX2. When looking at the time domain behaviour of this metric, it appears that the link is very unstable during working hours and fairly stable at night and in the weekends. In fact, the unstable periods correspond directly to those times during which the building where receiver RX3 was located and the buildings surrounding it (some low-rise offices and a very popular student restaurant) were full of people. This behaviour is very similar to the indoor link effects described in [17,51]. In these papers, significant signal degradation was observed on days when people were present inside the test building. As described in [17], these fluctuations are attributed to human body absorption. Remarkably, this is the first time that these effects are observed in an outdoor LoRa link. Although it appears that the 868 MHz link to RX3 is less impacted by this specific phenomenon, it can be observed that the measured signal levels fluctuate significantly more than along the other 868 MHz links.

The most interesting links are those to RX4. Due to the longer propagation distance, a lot more fluctuation is observed on these links. In fact, the most eye-catching features are the large signal drops occurring once or twice each day. They are also reflected in the standard deviation and PRR data in Table 4. This unusual behaviour will be investigated thoroughly in the next subsections. The link to RX5 was not active when the measurements presented in Figure 5 were gathered but, as will be shown in the next subsections, the node at RX5 detected similar signal fluctuations as registered by the node at RX4. As the power levels received by the nodes located closer to the transmitter show less fluctuations than those received by the long-distance nodes, these link variations are not significant enough for further statistical analysis.

### 3.2. Daily Signal Fluctuations on Long-Distance Links: General

**Figure 6:**
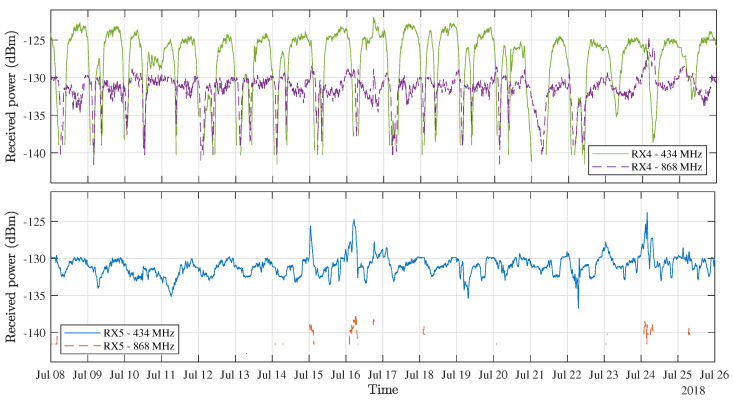
Selection of link monitoring data gathered at RX4 and RX5 during a heatwave in July 2018.

To enable a more rigorous characterisation of the large signal fluctuations found in the data gathered from the long-distance links to RX4, additional measurement campaigns were performed during the entire year that followed, employing the nodes at RX4 and RX5. This has resulted in a very large dataset, describing the performance of these long-distance links over a large variety of weather conditions. As a full time-domain representation of these data would be very impractical due to the large size of this dataset, only a selection of received power levels—more specifically those gathered in the month of July 2018—are shown in Figure 6. This time period was specifically chosen because of the very stable weather experienced in Belgium during those days. In fact, from 13–27 July, a heatwave was registered in the country, providing a sizeable stretch of days during which the potential influences of day-to-day variations of the weather conditions on these fluctuations were limited. In addition to this heatwave, hardly any rainfall was recorded for weeks on end during this period.

**Figure 7:**
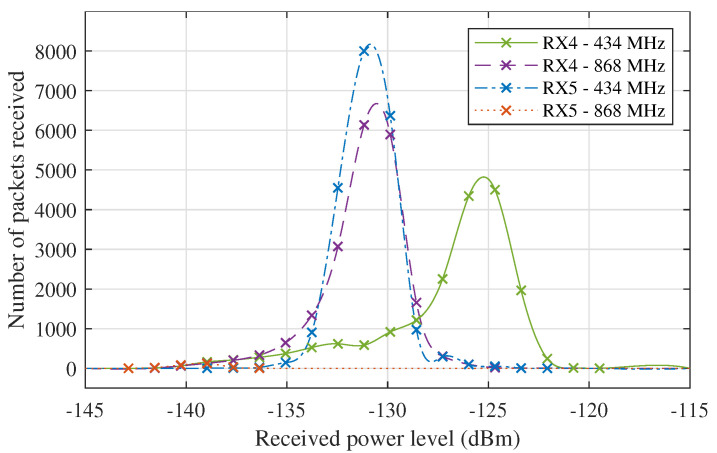
Received signal level distributions for the RX4 and RX5 data presented in Figure 6.

Figure 6 shows that the signal level fluctuations are very severe for the links to RX4, regularly lowering the signal level by over 15 dB for the 434 MHz link and by about 10 dB for the 868 MHz link. During these drops, the PRR practically reduces to zero, so the power loss could actually be even higher. For the 434 MHz links to RX5, the deteriorations are a lot less severe, as seen in these data’s standard deviation and PRR metrics that are presented in Table 5. Due to the large distance to the transmitter, the 868 MHz link to RX5 has a very low PRR, so little can be said about possible fluctuations in this link. Likewise, the standard deviation on the received power is very low for this link because of the extremely low SNR values that correspond to these received power levels. Based on the calibration data for the SNR measurements presented in [17], the background noise level is estimated at −114 dBm in both of the employed ISM bands. The influence of the signal degradations is also apparent in Figure 7, which shows the received signal level distributions obtained by performing piecewise polynomial interpolation on the histograms that describe the distribution of the received power levels presented in Figure 6. These distributions are reasonably symmetric in general, but the ones describing the RX4 data do have distinct irregular left sided tails, which are clearly caused by the signal deteriorations.

Although it is not possible to pinpoint the cause for these fluctuations upon this first observation, it is interesting to see that they occur in the two long-range links, while they are not observed in the data gathered by the nodes located closer to the transmitter or in the data gathered by the TX power monitor. Given that these degradations are indeed observed in both bands by both the RX4 and RX5 receivers and that they occur at different times every day, it can be ruled out that they would be caused by interference, either related to other ISM band users or to contributions of urban electromagnetic interference. The gradual recession and reappearance of the signal levels—with a time-constant larger than the window size of the moving-average filter used here—further supports this claim. In the long-distance links, the influence of interference is further reduced by the filtering that occurs in the LoRa receiver’s front-end, and by the application of the highly directional Yagi-Uda antennas, which are pointed towards the transmitter. Yet, to rigorously determine the source of the fluctuations shown in Figure 6, the data obtained using RX4 and RX5 will be analysed more deeply in the following subsections based on the correlation between the links (Section 3.3), the periodicity of the fluctuations (Section 3.4) and the influence of the weather on these occurrences (Section 3.5). Because of the low PRR registered using the 868 MHz link to RX5, this link will be omitted in these analyses.

### 3.3. Daily Signal Fluctuations: Correlation

First, it is interesting to look at the general correlation levels between the long-distance links under study. To this end, normalised correlation coefficients are calculated for all relevant data gathered using these three links (being those portions of the full dataset when both RX4 and RX5 were active). As shown in Table 6, they indicate a relatively weak positive correlation between the 434 MHz and 868 MHz data gathered at RX4. More peculiar is the slightly stronger, but negative correlation between the links to RX4 and the 434 MHz link to RX5. Despite their lower values, all of these coefficients are statistically significant with *p*-values smaller than 0.1%.

It is interesting to examine how these correlation levels change over time. To this end, the longest uninterrupted measurement campaign that took place at RX4 and RX5 is considered now. This link characterisation effort was carried out between 18 October 2018 and 5 May 2019. Descriptive statistics on the subsets of data gathered in this period are shown in Table 7.

**Figure 8:**
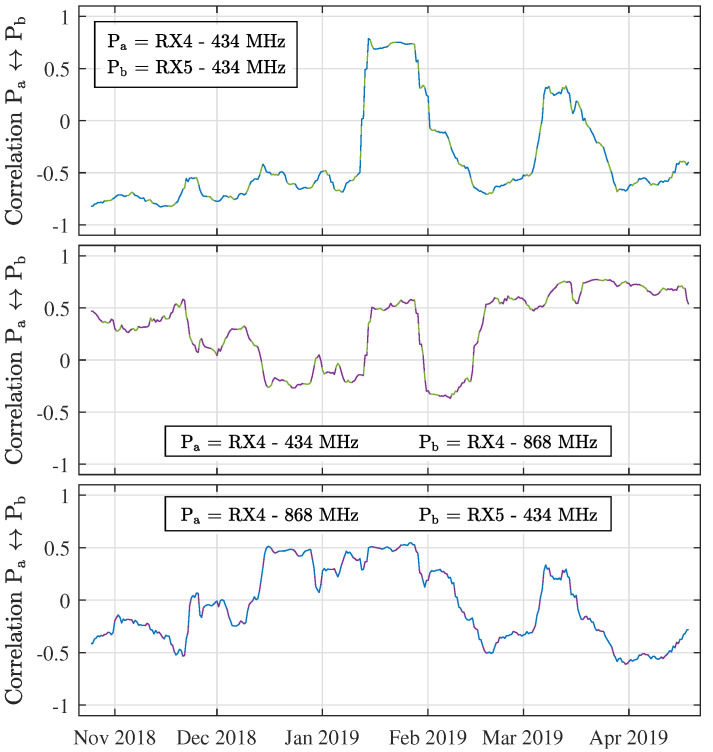
Normalized correlation between $P_{a}$ and $P_{b}$ over time (window = 14 days).

By daily sampling the correlation of both links for a time window of 14 days (which corresponds to 14×24×30= 10,080 measurements points), a high-resolution representation of how the correlation behaviour changes over time can be produced. Figure 8 shows the results of this operation for all three combinations of the three links under study. This figure primarily exposes that the correlation levels vary significantly over time and that they sometimes change polarity, which explains why the correlation values are fairly low in Table 7. This is very apparent for the power levels measured in the same frequency band ( 434 MHz) at different receiver locations (RX4 and RX5), as shown in the top part of Figure 8. In fact, absolute correlation levels between RX4 and RX5 are fairly high at 434 MHz. Interestingly, during the third week of January, when all three links are strongly and positively correlated with one another, several cm of snowfall were observed in Belgium. It is also observed that the correlation between both frequency bands (middle and bottom plots) tends to diminish during the rest of the winter, while near the end of autumn and throughout spring, these correlation levels are stronger, sometimes flipping between positive and negative levels. Additionally, these results hint at the influence of certain weather conditions on the presented link fluctuations. Consequently, this is investigated further in Section 3.5. However, first the strength of the periodicity of the link fluctuations is considered.

### 3.4. Daily Sgnal Fluctuations: Periodicity

**Figure 9:**
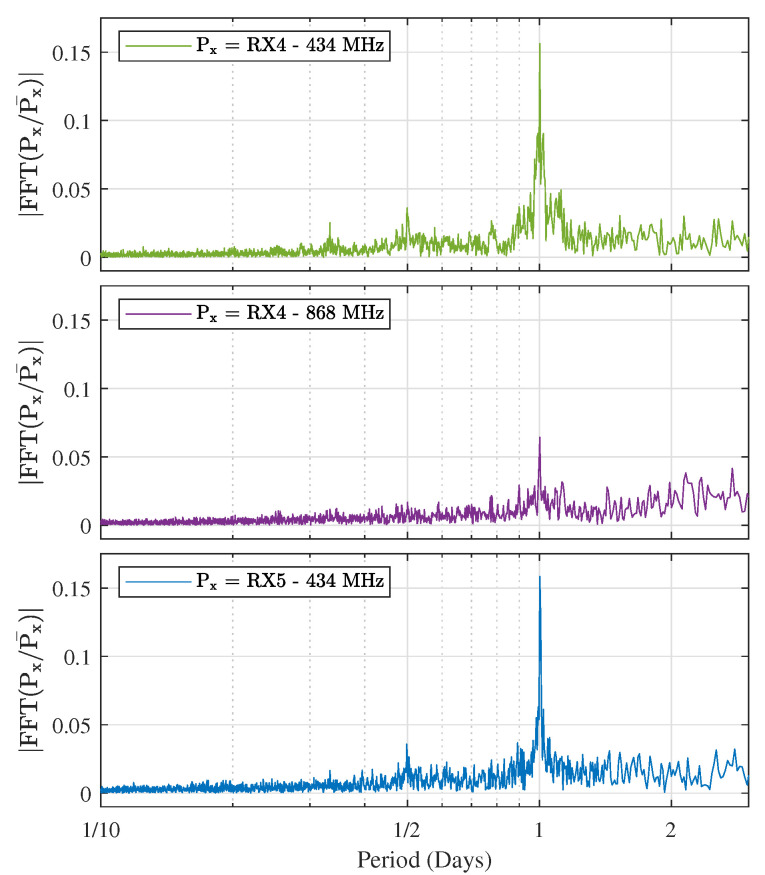
FFTs of the normalized linear power levels ($P_{x}/\bar{P_{x}}$) gathered by the receivers at RX4 and RX5.

To examine the periodicity of the signal fluctuations, the fast Fourier transform (FFT) was performed on the (linear) power levels gathered on the three long-distance links. To be able to compare the magnitude of certain peaks between different measurement locations, the measured powers were first normalized by dividing them by their mean value. Hence, the periodicity is calculated as $\left|\mathrm{FFT}(P_{x}/\bar{P_{x}})\right|$, where $P_{x}$ indicates the received power levels, $\bar{P_{x}}$ denotes the average received power and the subscript x indicates the receiver under study. The resulting graphs can be found in Figure 9. To make the result of the FFT-transformation easier to interpret, the frequency axis was inverted to show the period instead. Figure 9 shows very distinct peaks on the 1 day mark, proving that the fluctuations are recurring with a period of exactly one day, at both receiver locations. For the 434 MHz data, smaller peaks can also be observed at the half-day mark.

**Figure 10:**
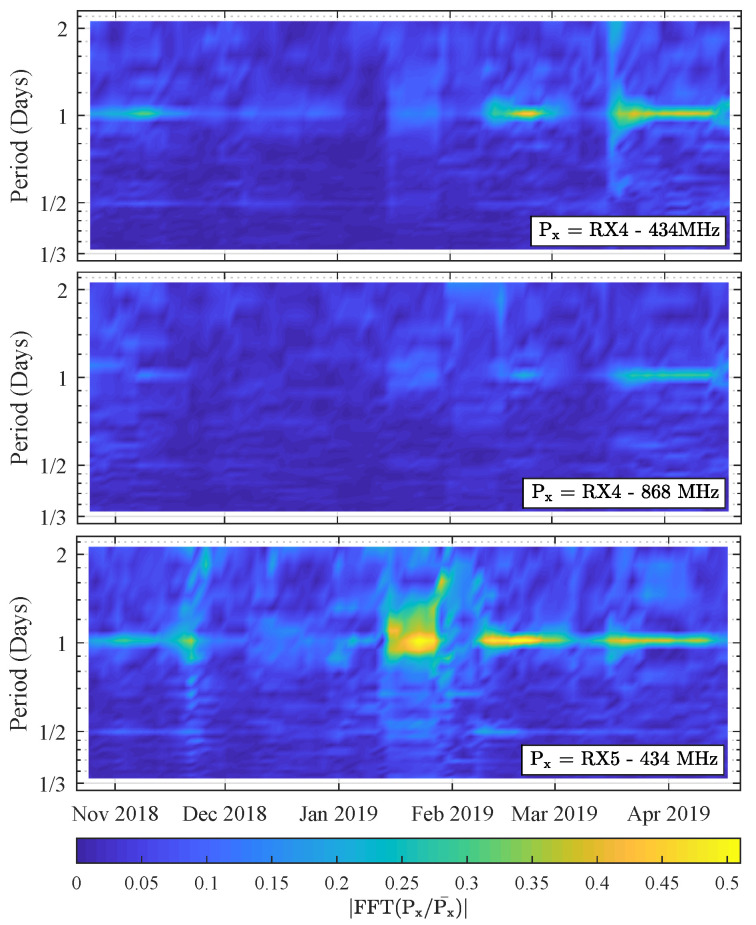
Spectrograms of the signal fluctuations observed at RX4 and RX5 (window = 14 days).

Additionally, it is also interesting to see how the FFT-profiles presented in Figure 9 change over time. To this end, these profiles were calculated each day with a window size of 14 days, resulting in the spectrograms shown in Figure 10. Again, a very clear trend is observable in all three of these plots: a distinct amount of energy is present on the one-day mark, near the end of autumn and during most of spring, which again hints at a weather-dependency of the signal fluctuations. In addition, during some of these moments, there is also a faint ridge on the half-day mark, resulting in the smaller peaks of Figure 9 that were discussed earlier. Furthermore, there are also some minor FFT-artifacts in these spectrograms, which were found to be caused by packet loss. This explains why the RX5— 434 MHz spectrogram is the noisiest.

**Figure 11:**
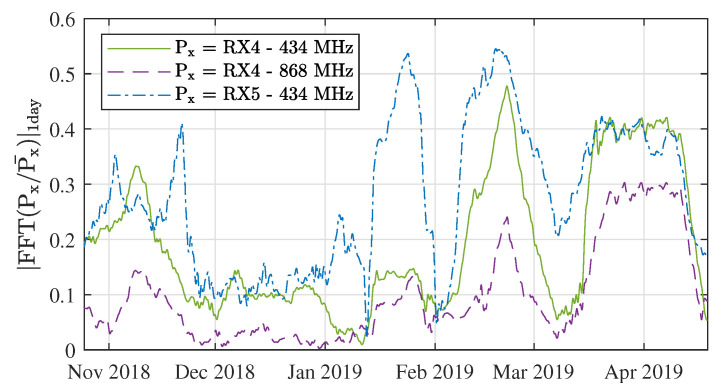
Intensity of the signal fluctuations over time, determined as the normalised energy in the FFT bins for period = 1 day in Figure 10.

As an extension to Figure 10, the amount of energy in the FFT bin corresponding to a period of one day is also shown in Figure 11. This value gives an indication of how the intensity of the signal fluctuations changes over time. As also shown in Table 8, there is a strong correlation between the intensity of the signal level fluctuations in all three links under study.

**Figure 12:**
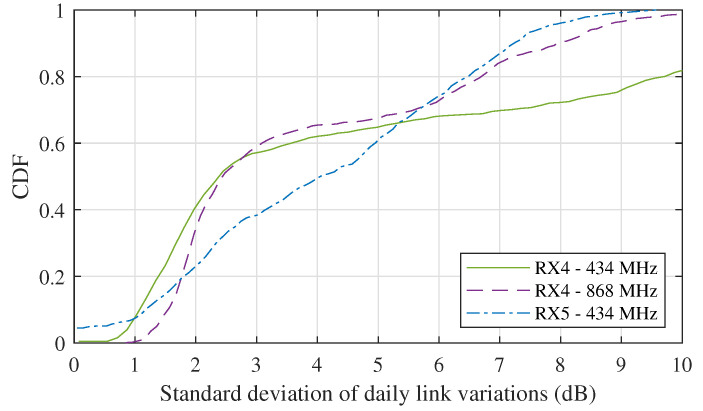
CDF of the standard deviations of the received power level fluctuations as measured daily for each link to RX4 and RX5.

Complementary to Figure 11, we can also assess the intensity of the fluctuations by considering the standard deviation of the links on a daily basis. For the measurement campaign described in Figure 11, these standard deviations are compiled in a cumulative density function (CDF) to give an indication of the percentage of time in which certain fluctuations were observed. The result, shown in Figure 12, shows that for the RX4 links, the standard deviation of these fluctuations is limited to 3 dB in 60% of the days in the measurement campaign. For the 434 MHz link to RX5, this is the case for 40% of time. Furthermore, for approximately 75% of the days, the standard deviation stayed under 6 dB at both receiver locations. Yet, more extreme variations up to 10 dB did sporadically occur.

### 3.5. Daily Signal Fluctuations: Influence by Weather

**Figure 13:**
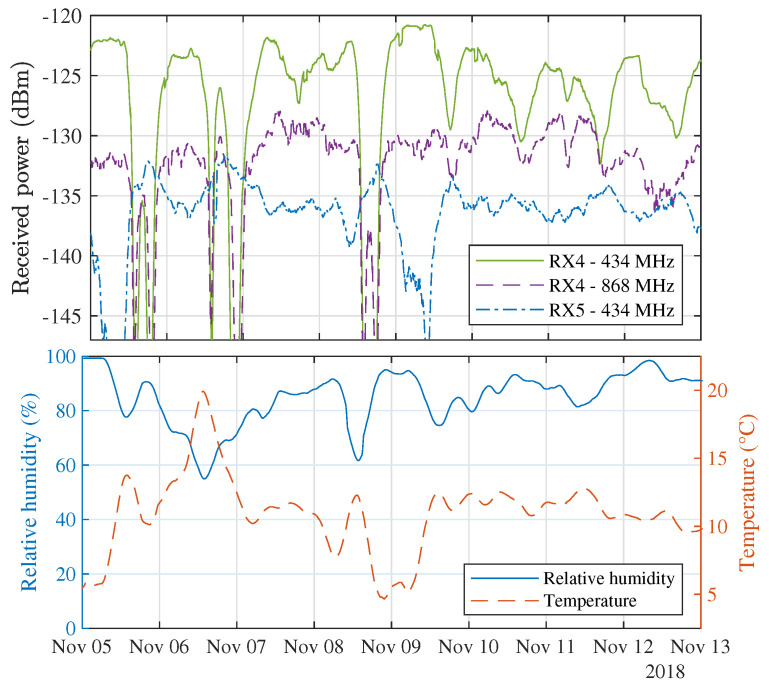
Power levels received from 5 November until 12 November 2018 (**top**) and the ambient temperature and relative humidity during those days (**bottom**).

Throughout the entire year, many co-occurrences between weather phenomena and signal fluctuations were observed. A specific example of this is shown in Figure 13. Here, the power levels gathered during 8 days in the first half of November 2018 are considered along with the relative humidity and temperature at that time, as provided by the public observatory Armand Pien of Ghent University and part of the dataset presented in [52]. Over the course of these 8 days, the weather was relatively unstable, which manifested itself in the absence of large temperature and humidity variations on certain days. Interestingly, on 7, 10, 11 and 12 November, the absence of temperature and humidity variations directly coincides with less severe drops in signal level.

**Figure 14:**
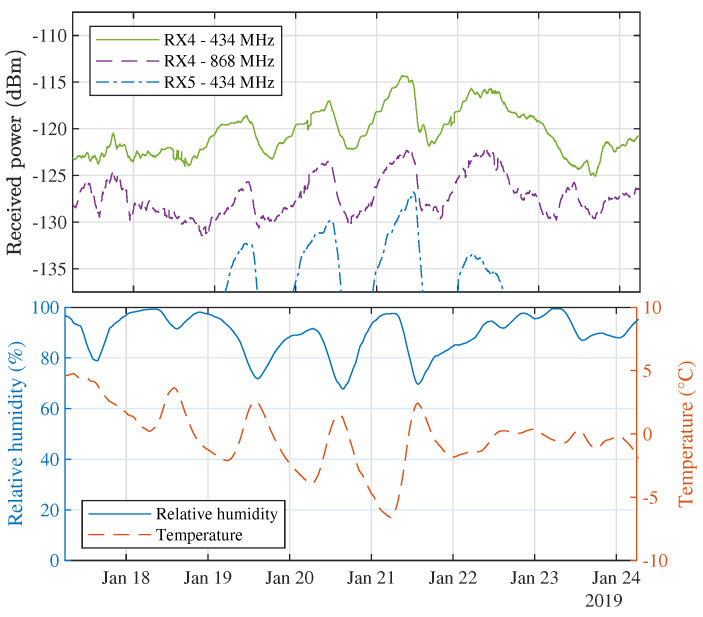
Power levels received during several days of episodic light snowfall in January 2019 (**top**) and the ambient temperature and relative humidity during those days (**bottom**).

It is also interesting to zoom in on the snowfall that occurred near the end of January, as mentioned in Section 3.3. In all three long-distance links, this snowfall resulted in very strong periodic signal fluctuations and significant signal strength enhancements. The power levels received during these days are shown in Figure 14, where they are compared to the ambient temperature and relative humidity at that time. This figure clearly demonstrates how the received power levels are distinctly dependent on the weather conditions that were observed when these data were gathered. However, as was shown in Section 3.3, most of the data that were gathered are less correlated across the different receiver locations than during these three days in January. Moreover, for the majority of the measurements, an inverse correlation was found between RX4 and RX5 at 434 MHz.

To assess the general weather dependency of the signal fluctuations, larger sets of meteorological data need to be examined as well. In order to provide a general indication of the correlation between the ambient temperature and humidity data and the received power levels, the correlation coefficients between these measures are given in Table 9. First of all, these numbers show that the data gathered at RX5 is strongly correlated with the ambient temperature. Because of the very strong negative correlation between the temperature and humidity, this results in a moderately strong correlation with the relative humidity as well. Additionally, Table 9 shows a weaker—yet still statistically significant—correlation between the RX4 data and the weather parameters under study. Once more, there is a disparity between the signs of the correlation values.

**Figure 15:**
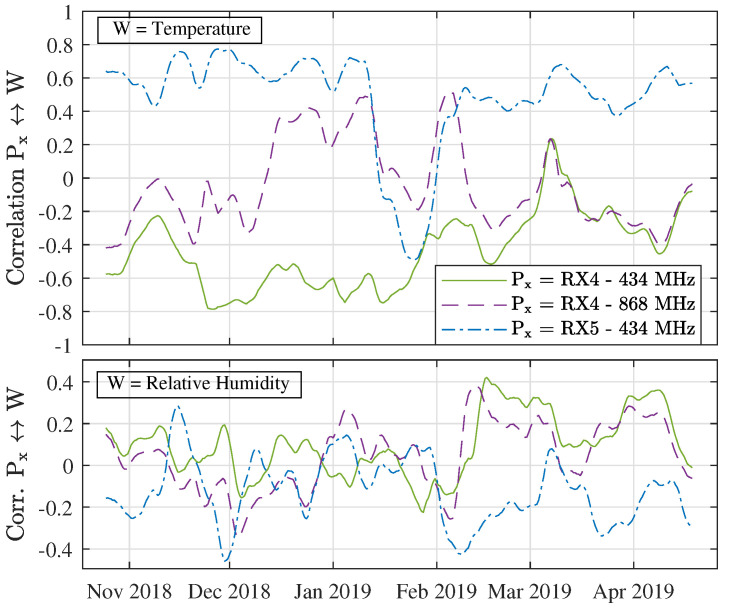
Normalized correlation coefficients between the received power levels ($P_{x}$) and ambient temperature (**top**) or relative humidity (**bottom**) over time.

Again, a lot more can be learned from looking at how these correlation levels change over time. Therefore, the variation of these correlation levels is shown in Figure 15. This figure shows the 14-day moving average correlation between the received power levels and the weather parameters under study. Hence, for each day in the measurement campaign, the correlation between the received power and the temperature/humidity are calculated for the past fourteen days. In order to increase the readability of the graph, an additional moving average filter with a window size of 7 days was used as well. Figure 15 mainly shows that the correlation between the signal fluctuations and the weather parameters under study is the strongest for the 434 MHz links. Interestingly, the correlation between the data gathered at RX5 and the temperature is strong and positive during the entire measurement campaign except around those days when snowfall was recorded (see also Figure 14), when it suddenly becomes negative. In general, the correlation behaviour between the received power levels and the relative humidity is less convincing as the relevant correlation coefficients are lower and fluctuate more over time.

**Figure 16:**
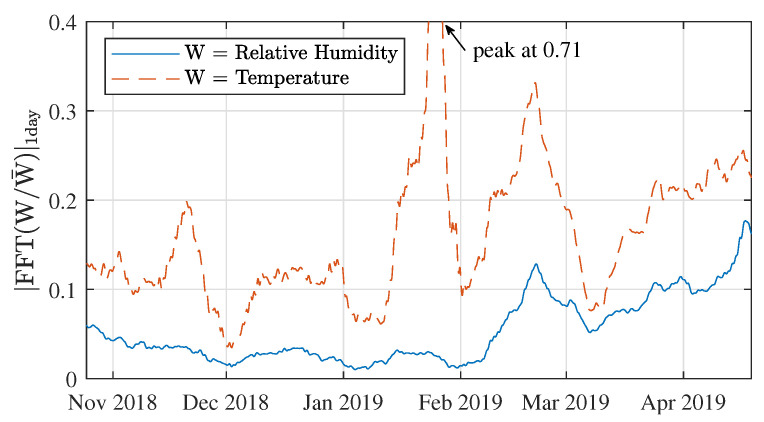
Intensity of the weather fluctuations over time, determined as the normalised energy in the FFT bins for period = 1 day, when describing the frequency content of the ambient temperature and relative humidity data.

As a final step in this analysis, the strength of the temperature and humidity variations is displayed as a function of time in Figure 16. This figure was made in the same way as Figure 11, with the exception that the intermediate step of showing spectrograms first is skipped here. Ultimately, given the great similarity between this figure and Figure 11, this is the best example of how the periodic signal fluctuations presented in this work are strongly connected to the weather. It shows that greater temperature and humidity variations directly correspond to heavier signal drops, which is also reflected in the correlation coefficients that describe this correspondence in Table 10.

## 4. Discussion

Based on the evidence presented in Section 3, it is clear that the daily signal deteriorations presented in this work are at least partly caused by weather-related effects. When trying to identify potential contributions of certain weather-related tropospheric propagation effects, a few well-known large-scale effects and mechanisms can be eliminated right away. Scattering by gaseous structures in the direct link path, attenuation due to absorption by gaseous structures and attenuation due to precipitation are unlikely to be direct causes of this phenomenon as literature suggests that these mechanisms only have a noticeable influence at higher frequencies [27,53,54,55]. Furthermore, potential contributions from tropospheric scintillations are eliminated from the data by the moving average process mentioned in Section 3.1. Since the receivers were all located well within the radio horizon of the transmitter (which is approximately equal to 30 km for this transmitter setup), diffraction on the earth itself is not relevant here either.

However, diffraction around static or quasi-static objects such as buildings, infrastructure and vegetation may be playing a role in this phenomenon. In fact, the weather-dependency of the signal degradations indicates that daily variations in tropospheric refractivity may contribute to the signal fluctuations; and as is mentioned in [56,57], these changes in refractivity can have an influence on the severity of the fading process caused by diffraction around the static and quasi-static obstructions mentioned earlier. This obstruction fading process would also explain why the signal degradations measured by RX4 and RX5 seem somewhat different, as each link is unique, having its own dominant obstructions. Additionally, as tropospheric refractivity variations may alter the various link paths in different ways, diverse multipath contributions can be expected to impact the performance of the links differently as well. Furthermore, a contribution of troposcatter cannot be ruled out as this mechanism is present in any tropospheric radio link.

In addition to causing certain propagation effects, seasonal weather effects may also have an impact on the performance and reliability of communication hardware. For example, outdoor antennas may suffer from the proximity of water or other precipitation such as snow and ice. In fact, in [17], decreased antenna performance was observed for a wireless link that employed an outdoor antenna during a period of significant rain, while the signal received by an indoor antenna remained largely unimpacted. Finally, extreme temperatures and temperature swings may impact the noise performance of RF-circuitry and power delivery circuitry in exposed IoT hardware. Yet, as presented in [31,32], the received power variations that are expected to result from this mechanism are an order of magnitude smaller than the link variations presented in this work. Moreover, as the correlation between the ambient temperature and received power levels presented here is relatively variable, it is clear that propagation effects are strongly dominant.

In the past, effects similar to the signal deteriorations and enhancements presented in this work have been observed and documented for some very long-distance links, generally using higher-altitude antennas at both sides of the links. Most research on this topic concentrates on tropospheric ducting in over-sea paths as this is where super-refractive phenomena occur most often and are most pronounced [58,59,60,61,62]. In [63,64], the link was made between signal strength enhancements and the presence of anticyclones, which causes low-altitude tropospheric stratifications by means of subsidence and advection, resulting in anomalous propagation conditions [58,65,66]. Furthermore, in more recent link characterisation research, it was shown that snowfall has a detrimental effect on link quality [33] and that packet reception is slightly lower in summer months when compared to winter months [41].

With the advent of the IoT and 5G, new long-range, low-power technologies are being developed for which the variable link performance presented in this work and propagation effects presented elsewhere may be very relevant. Not only could these large signal deteriorations cause coverage reduction in these networks, possible increases in range may also cause interference between neighbouring networks. As a result, this may severely lower the quality of service (QoS) for those LPWAN network technologies such as LoRa for which the scalability is already being questioned [6,7,29,30]. Therefore, knowledge about relevant weather-related propagation phenomena should be updated for modern day environments such as large urban, suburban and industrial areas, considering their importance in long-range sensor networks and the IoT, as well as for natural environments where IoT-based monitoring solutions can support initiatives aiming to increase the sustainability of modern society.

A distinction can be made between frequent link fluctuations, caused by variations in refractivity and diffraction around obstacles, ever changing multipath conditions, etc. and infrequent fluctuations, such as those caused by snowfall or extreme heat. Wether either of these types of fluctuations are problematic for a given network setup is to be determined by the designer, based on the characteristics of each network. For example, for sensor communication systems, a link outage may be tolerable, while it might be unacceptable when sharing time-critical information. Figure 11 and Figure 12 may aid the system designer in their analysis.

## 5. Conclusions

Custom low-power channel characterisation hardware was used to monitor the performance of a (sub)urban LoRa network featuring 10 outdoor wireless links communicating over both the 434 MHz and 868 MHz ISM-bands. A lot of attention went to the performance of two long-distance links spanning 10.6 km and 13.9 km on which large signal deteriorations were observed. These > 15 dB signal drops were found to be correlated significantly between both frequency bands and both receiver locations. They were also found to be periodic with a period of exactly one day, additionally showing some half-day periodicity during certain weather conditions. Upon further analysis, multiple co-occurrences were observed between certain weather conditions and the shape and regularity of the signal deteriorations. The strongest evidence for this relation between the signal fluctuations and the weather is undoubtedly the strong correlation between the intensity of the weather variations and the severity of the signal fluctuations.

Mechanisms causing these signal fluctuations may be tropospheric in nature. More specifically, received power levels may vary due to daily variations in the refractivity of the troposphere, which in turn may have an influence on the fading process caused by diffraction around buildings, infrastructure and vegetation on the link path. Additionally, possible contributions of troposcatter and multipath cannot be ruled out. Finally, diminished antenna performance due to humidity or reduced hardware performance due to temperature fluctuations may also contribute to these effects. Keeping in mind the severity of the signal deteriorations described in this work, it is clear that both frequent as infrequent weather-induced link deteriorations should be taken into account when designing IoT-networks in order to increase their quality and reliability.

## Figures and Tables

**Table 1:** Maximum radius ($r_{1}$) of the first Fresnel zone and indications of the minimum clearance of this zone when assuming that no obstacles are present ($c_{0}$). In reality, a lot of obstacles are also present on the Earth’s surface, which further reduce the first Fresnel zone’s clearance.

	$r_{1}$ (m)		$c_{0}$ (%)	
	**434 MHz**	**868 MHz**	**434 MHz**	**868 MHz**
RX1	19.0	13.5	100	100
RX2	26.3	18.6	69.5	98.3
RX3	26.3	18.6	54.3	76.8
RX4	42.8	30.3	49.9	70.6
RX5	49.0	34.6	41.1	58.2

**Table 2:** Packet structure. TX ID: transmitter identifier string, VDD: supply voltage, TEMP: ambient temperature.

TX ID	TIMESTAMP	PACKET N°	VDD	TEMP
4 bytes	6 bytes	2 bytes	2 bytes	2 bytes

**Table 3:** LoRa modulation and general network settings.

Parameter	Value
Transmit power	10 dBm
LoRa spreading factor (SF)	12
LoRa bandwidth (BW)	125 kHz
LoRa coding rate (CR)	4/5
Bit rate	293 bps
Packet length	16 bytes
Packet rate	1 packet/2 min

**Table 4:** Descriptive statistics on the general performance of the LoRa network links (as registered in May 2018).

	434 MHz			868 MHz		
	μ **(dBm)**	σ **(dB)**	PRR **(%)**	μ **(dBm)**	σ **(dB)**	PRR **(%)**
RX1	−82.6	1.5	100	−99.2	1.7	96.0
RX2	−99.5	1.9	99.1	−121.3	2.1	97.0
RX3	−112.3	5.0	98.6	−123.0	4.6	97.6
RX4	−128.7	5.1	82.0	−131.4	3.3	86.3
RX5	Not yet active.					

**Table 5:** Descriptive statistics on the performance of the links to RX4 and RX5 during a heatwave in July 2019.

	434 MHz			868 MHz		
	μ **(dBm)**	σ **(dB)**	PRR **(%)**	μ **(dBm)**	σ **(dB)**	PRR **(%)**
RX4	−127.1	3.6	85.0	−131.2	2.1	92.6
RX5	−131.0	1.43	99.7	−139.4	1.1	1.2

**Table 6:** Normalised correlation coefficients between power levels received over the long-distance links.

		RX4		RX5
		**434 MHz**	**868 MHz**	**434 MHz**
RX4	434 MHz	1	0.2045	−0.2308
	868 MHz	0.2045	1	−0.2788
RX5	434 MHz	−0.2308	−0.2788	1

**Table 7:** Performance of the links to RX4 and RX5 between 18 October 2018 and 5 May 2019.

	434 MHz			868 MHz		
	μ (dBm)	σ (dB)	PRR (%)	μ (dBm)	σ (dB)	PRR (%)
RX4	−123.4	4.4	93.0	−129.0	3.1	93.6
RX5	−136.6	3.23	62.1	N/A	N/A	<0.001

**Table 8:** Normalised correlation coefficients between the periodicity of the fluctuations registered at RX4 and RX5.

		RX4		RX5
		**434 MHz**	**868 MHz**	**434 MHz**
RX4	434 MHz	1	0.8907	0.6775
	868 MHz	0.8907	1	0.6258
RX5	434 MHz	0.6775	0.6258	1

**Table 9:** Normalised correlation coefficients between the received power levels and the ambient temperature and relative humidity.

		Temperature	Humidity
RX4	434 MHz	−0.3226	0.1259
	868 MHz	−0.3040	0.2272
RX5	434 MHz	0.6509	−0.4497

**Table 10:** Normalised correlation coefficients between the intensity of the signal fluctuations (as shown in Figure 11) and the intensity of the variations in ambient temperature and relative humidity (as shown in Figure 16).

		Temperature	Humidity
RX4	434 MHz	0.4175	0.7046
	868 MHz	0.4846	0.7220
RX5	434 MHz	0.7305	0.5019

## Data Availability

Not applicable.

## Abbreviations

The following abbreviations are used in this manuscript:

IoT	Internet of Things
WSN	Wireless sensor network
LPWAN	Low-power wide-area network
NB-IoT	NarrowBand IoT
LTE-M	Long Term Evolution-Machine Type Communication
PCB	Printed Circuit Board
IMU	Inertial Measurement Unit
SNR	Signal-to-Noise Ratio
PVC	Polyvinyl chloride
TX	Transmitter
RTC	Real-Time Clock
RX1	Receiver 1
RX2	Receiver 2
RX3	Receiver 3
RX4	Receiver 4
RX5	Receiver 5
LoS	Line-of-Sight
NLoS	Non-Line-of-Sight
nLoS	Near-Line-of-Sight
PRR	Packet reception ratio
ISM	Industrial, scientific and medical
TX ID	Transmitter identification
VDD	Power supply level
TEMP	Ambient temperature
SF	Spreading Factor
BW	Bandwidth
FFT	Fast fourier transform
QoS	Qualiy of service
